# Prevalence and Morphological Characterization of* Cheilospirura hamulosa*, Diesing, 1861 (Nematoda: Acuarioidea), from Partridges in Iran

**DOI:** 10.1155/2015/569340

**Published:** 2015-11-26

**Authors:** Maryam Ebrahimi, Soheila Rouhani, Iraj Mobedi, Ali Rostami, Hoshang Khazan, Mohammad Bagher Ahoo

**Affiliations:** ^1^Department of Parasitology and Mycology, School of Medicine, Shahid Beheshti University of Medical Sciences, Tehran, Iran; ^2^Department of Medical Parasitology and Mycology, School of Public Health, Tehran University of Medical Sciences, Tehran, Iran; ^3^Department of Parasitology, Faculty of Veterinary Medicine, University of Tehran, Tehran, Iran

## Abstract

This study reports data on the prevalence, morphology, and morphometry of the nematode* Cheilospirura hamulosa* on the basis of light and stereoscopic microscopy and also camera lucida. Specimens were recovered after necropsies of 100 partridges (*Alectoris chukar*) from Taleqan County in Alborz Province, Iran. The prevalence of* C. hamulosa* in partridges was of 30% with a mean intensity of 3.9 and range of infection of 1–12. The mean length and width of females were 17.5 ± 2.14 and 0.39 ± 0.04 mm, while those of males were 12.2 ± 0.67 and 0.3 ± 0.06 mm, respectively. The characteristic digitiform tail was observed in females, and the unequal spicules, caudal alae, and ten pairs of caudal papillae were seen in males. The taxonomic characteristic longitudinal cordons and muscular and glandular oesophagus were observed in both sexes. Ratio between cordons and body length in males and females was 1 : 1.33 and 1 : 1.68, respectively. Ratio between long and short spicules in males was 1 : 2.3. The average size of embryonated eggs was 51.25 × 29.5 *μ*m. In the present study,* C. hamulosa* (Nematoda: Acuarioidea) is recorded for the first time from partridges in Iran. Therefore, the morphological characters described in this study will be useful in the future diagnostic and taxonomic studies of Acuarioidea family.

## 1. Introduction

The parasitic nematode* Cheilospirura hamulosa*, “Diesing, 1861” (syn.* Acuaria hamulosa*), is a species of Acuariidae family and etiological agent of cheilospirurosis in birds such as chickens, turkeys, pigeons, and guinea fowls [1, 2].* Cheilospirura hamulosa *has cylindrical body with two triangular lips and 4 cuticular cordons that extend near posterior extremity. Male worms have two distinctly unequal and dissimilar spicules. Female worm tails are curved ventrally and digitiform [3, 4].

The* C. hamulosa *is located under gizzard cuticle mainly in koilin or muscular wall of the host [1, 2]. This nematode has indirect life cycle. The grasshoppers (*Melanoplus*,* Oxyanitidula*, and* Spathosternum parasinifrum*), beetles, and weevils are intermediate hosts for* C. hamulosa* and birds acquire their infections by eating contaminated arthropods containing infective third-stage larvae [1]. Due to its high pathogenicity in poultry, the* C. hamulosa* has veterinary and public health importance.* Cheilospirura hamulosa *can cause several complications such as granulomas and nodules that lead to anemia, impotence, and mortality in chickens [2].* Cheilospirura hamulosa *may cause zoonosis and has been recovered from a nodule on the conjunctiva of a Filipino farmer [5]. The previous studies in rural areas of Iran indicated that the infection of this worm is common among free-range chickens [6], whereas there is no published data on partridges in Iran. The main goal of the present study was to survey prevalence of* Cheilospirura hamulosa* infection in partridges (*Alectoris chukar*) using morphological-based methods, in Taleqan County of Iran.

## 2. Materials and Methods

The study was conducted in Taleqan County. This area is located in Alborz Province and its height is 1900 m above the sea level. Latitude and longitude of Taleqan are 36 degrees 15′N and 50 degrees 46′E, respectively.

One hundred partridges were collected from Taleqan mountainous region between 2011 and 2013. Every year from the end of September to the beginning of February a license is issued by the Environment Protection Agency (EPA) of Iran for hunting partridges. Every hunter has permission to hunt the maximum of three partridges weekly. For this research, the gizzard of each partridge was removed from the alimentary tract and delivered to the helminthology laboratory of Shahid Beheshti University of Medical Sciences. The gizzards were examined macroscopically and then dissected in a 0.85% NaCl solution (normal saline) to remove cuticle. Worms were visible to the naked eye. The worms were rinsed in normal saline and fixed in Alcohol-Glycerin (70% Alcohol, 50 mL; Glycerin, 50 mL) solution. Nematodes were elucidated with acetic acid and phenol, mounted in Canada balsam. Helminthes were counted and identified under light microscopy (Zeiss, Germany) and stereoscopic microscope (Zeiss, Germany) and traced by camera lucida (Zeiss, Germany). The morphological identification of the nematodes to the species level was done according to methods described by Skrjabin et al. [9].

## 3. Results

### 3.1. Prevalence of* C. hamulosa*

**Figure 1:**
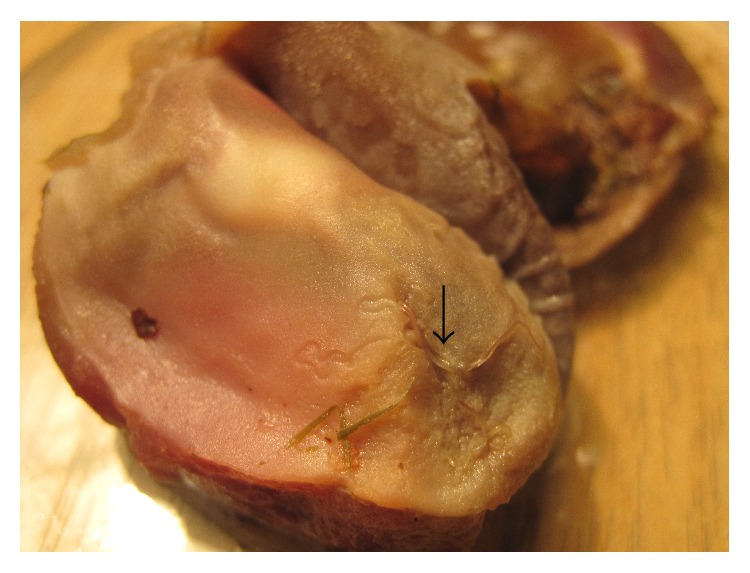
*Cheilospirura hamulosa* under the gizzard cuticle.

Out of a total of 100 partridges examined, the prevalence of* C. hamulosa *was 30% with a mean intensity of 3.9% and range of infection of 1–12. A total of 116 worms were recovered from partridges. Among the recovered helminthes, 60.3% were female and 39.7% male. The specimens of* C. hamulosa *were found free under the gizzard cuticle, partially or fully burrowed in the walls (Figure 1). Microscopic description was based on 10 adult worms, five males and five females (Table 1).

### 3.2. Male (*n* = 5, Except When Otherwise Indicated)

**Figure 2:**
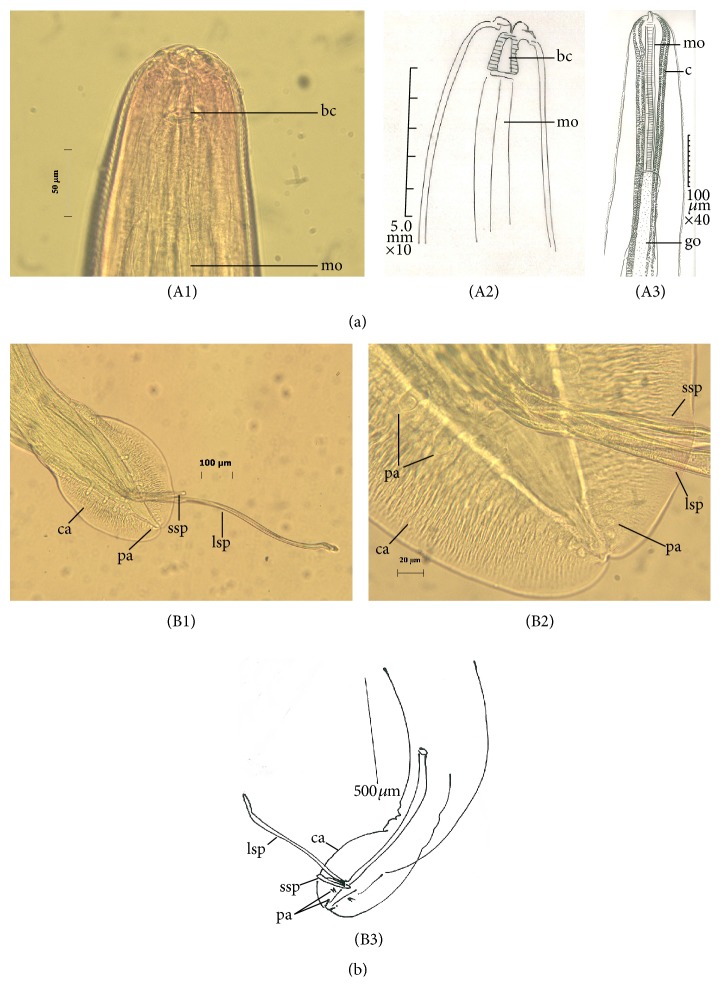
Male of* Cheilospirura hamulosa*; light micrographs and camera lucida. (A1) Enlarged view of the anterior region, buccal cavity (bc), muscular oesophagus (mo); (A2) anterior end, lateral view, buccal cavity (bc), muscular oesophagus (mo); (A3) anterior end, sublateral view, muscular oesophagus (mo), glandular oesophagus (go), and cordon (c); (B1) and (B2) male posterior end, showing small (ssp) and large (lsp) spicules, caudal alae (ca), and caudal papillae (pa); (B3) posterior end of male, lateral view, papillae (pa), long spicule (lsp), short spicule (ssp), and caudal alae (ca).

The average length of adult males was 12.2 ± 0.67 mm and their average width was 0.3 ± 0.06. Buccal cavity was 0.21 long, 0.019 wide (*n* = 1). Muscular oesophagus was 0.37–0.38 (0.375, *n* = 2) long. Glandular oesophagus was 1.6–2.4 (2, *n* = 2) long. Length of total oesophagus was 1.97–2.78 (2.37, *n* = 2). The mean of cordons length was 9.2 ± 0.28, ratio between cordons and body length was 1 : 1.33 (Figure 2, (A1)–(A3)), long spicule slender was 1.44 ± 0.08 mm in length, short spicule shaped like a chopping knife was 0.62 ± 0.11 mm long, and ratio between long and short spicules was 1 : 2.3. The ratio between mean of long and short spicules length and body length were 0.11 mm and 0.04 mm, respectively. The mean length of caudal alae was 0.42 ± 0.02 and its width was 0.32 ± 0.02, with tail 0.43 mm long (*n* = 1). In each worm there were ten pairs of caudal papillae: three couples were observed in precloacal, two pairs in adcloacal, and five couples in postcloacal (Figure 2, (B1)–(B3)).

### 3.3. Female (*n* = 5, Except When Otherwise Indicated)

**Figure 3:**
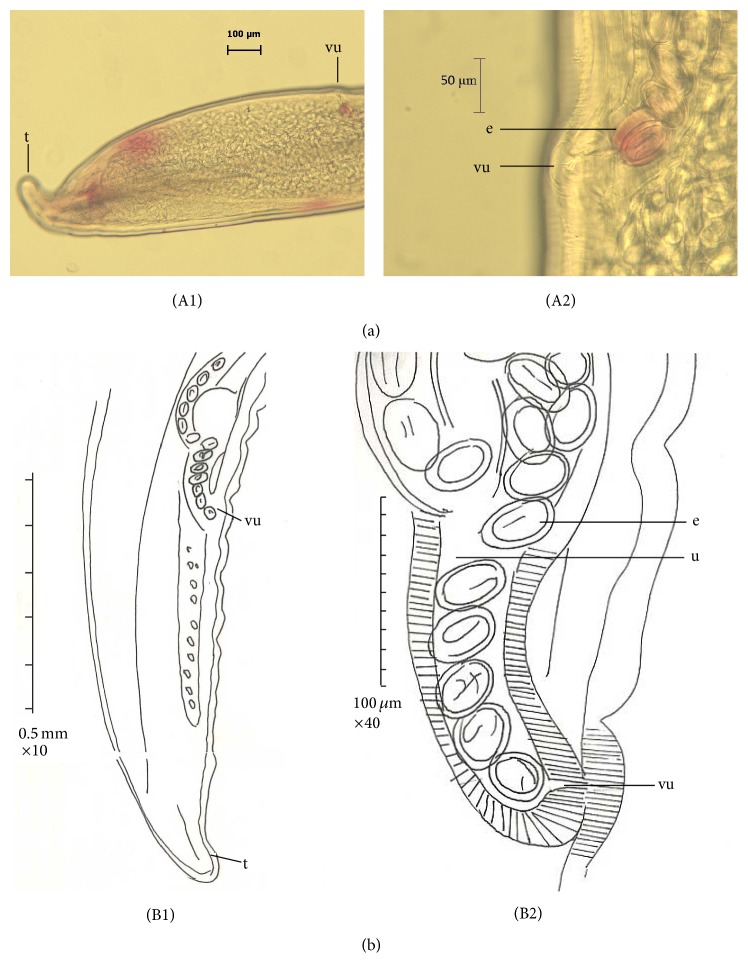
Female of* Cheilospirura hamulosa*; light micrographs and camera lucida. (A1) Female posterior end, showing vulva (vu), tail (t); (A2) enlarged view of the female posterior region, showing vulva (vu), embryonated eggs (e); (B1) and (B2) posterior end of female, vulva (vu), utri (u), egg (e), and tail (t).

**Figure 4:**
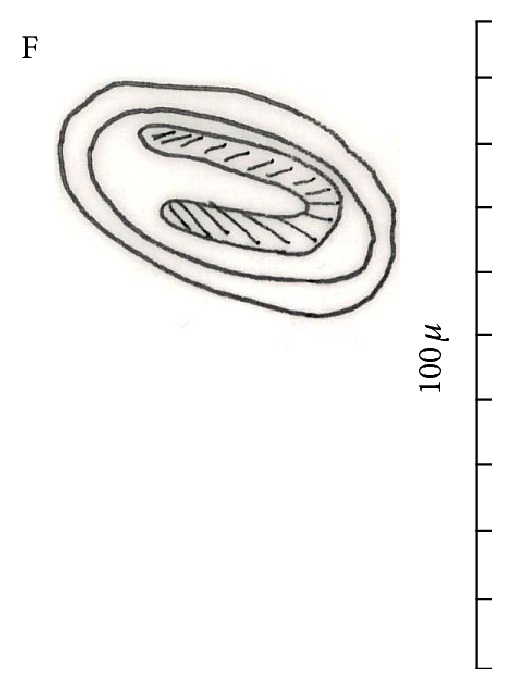
Camera lucida of embryonated egg.

The mean length and width of females were 17.5 ± 2.14 and 0.39 ± 0.04 mm, respectively. Buccal cavity was 0.22 long and 0.028 wide (*n* = 1). Muscular oesophagus was 0.29–0.75 (0.45, *n* = 3) long. Glandular oesophagus was 2.1–2.7 (2.3, *n* = 3) long. Length of total oesophagus was 2.39–3.05 (2.82, *n* = 3). The mean of cordon length was 12.96 ± 0.72, and ratio between cordons and body length was 1 : 1.68. The females were amphidelphic, and their vulva is located slightly posterior to the middle of the body at 2.29 ± 0.92 from the posterior end, circular sphincter 0.064 × 0.065, tail 0.44 (*n* = 1) long (Figure 3, (A1)-(A2) and (B1)-(B2)). Embryonated eggs were 0.045–0.055 (0.051) long and 0.028–0.03 (0.029) wide (Figure 4).

## 4. Discussion

The nematode* C. hamulosa* reported herein is reported for the first time from partridges in Iran. Studies on this nematode in Iran only was carried out in native fowls. The partridge (*Alectoris chukar*) is the most important bird hunted in Iran. In the present study, prevalence of* C. hamulosa* in the partridge was 30% with a mean intensity of 3.9% and range of infection of 1–12.

The reported prevalence of this nematode by Menezes et al. [2] in Brazil was 14.3% in ring-necked pheasants (*Phasianus colchicus*) with a mean intensity and range of infection of 1.5, 1-2, respectively. In domestic chickens (*Gallus g. domesticus*), the prevalence, mean intensity, and range of infection were 26.7%, 4 and 1–12, respectively [2]. A 2-year study in Kashmir, India, on the prevalence of the nematode* C. hamulosa *in indigenous fowl has shown an overall prevalence of 3.5% (17/478) [10]. The prevalence of* C. hamulosa* in guinea fowls (*Numida meleagris galeata *Pallas) from Ghana and chickens in Zimbabwe and Cuba was 37.8%, 46.6%, and 84.6%, respectively [11–13]. These results are higher than our finding. The reported prevalence of* C. hamulosa* on native fowls from Golestan Province in north of Iran was 4% which is significantly lower than our result, because the study population is completely different from our study [6].

The nematodes of the genus* Acuaria* (*Cheilospirura*) are parasitic among different families of birds and they are located under the koilin layer usually in the cardiac or pyloric regions [2]. Also in our study the specimens of* C. hamulosa* were found under the gizzard cuticle, partially or fully burrowed in the walls of the organ (Figure 1).

Most of the reported lengths for male and female* C. hamulosa* are within the range of 9–14 and 15–25, respectively ([3, 14] and [1, 7, 8]) (Table 1). In this study, male worms were smaller than the female worms in average body length, overall cordon, glandular oesophagus, and muscular oesophagus length (Table 1). Moreover, in our study, the long spicules were smaller and the length of short spicules was longer compared with previous studies reported by Cram [1, 3], Yamaguti [7], and Gomes et al. [8] (see Table 1 for comparison). In our study, average length of male worms was higher than those reported by Gomes et al. [8]. In our study, the female worms were smaller in maximum body length than those described by Cram and Yamaguti [3, 7] but still were bigger than those described by Gomes et al. [8]. The female of* C. hamulosa* in our study had smaller muscular and glandular oesophagus than those reported by Gomes and Yamaguti [7, 8], whereas values obtained for the length of tail and egg size were bigger compared to two studies mentioned above (Table 1). We observed 10 pairs of papillae in* C. hamulosa *as reported by Cram [3] and Gomes et al. [8]; however, in the precloacal region, 2 pairs of papillae were unclear (Figure 2, (B1)–(B3)).

Due to the significant pathogenic effects of these nematodes (*Cheilospirura *spp.) in poultry and wild bird population and very limited prevalence data of these helminthes in Iran, further study will be needed on different aspects of Acuarioidea family including pathogenesis and their prevalence in other avian species.

## 5. Conclusions

In the present study, we report for the first time the isolation and morphological characterization of* Cheilospirura hamulosa *from partridges in Iran. The morphological characters described in this study will be useful in the future diagnostic and taxonomic studies of Acuarioidea family.

## Figures and Tables

**Table 1:** Comparison of *Cheilospirura hamulosa* body parts from partridges of Iran with those of previous reports (mm).

	Cram, 1931 [1]	Yamaguti, 1954 [7]	Gomes et al., 2004 [8]	This study, 2014
*Male*	—	*n* = 1	*n* = 4	*n* = 5
Body, length (mm)	9–13	13.6	9.54	11.18–13 (12.22)
Body, width (mm)	0.3–0.32	0.32	0.28	0.25–0.4 (0.3)
Cordons, length	7.2–8.8	—	—	9–9.6 (9.2)
Buccal cavity	—	0.24 × 0.03	—	0.21 × 0.019^*∗*^
Muscular oesophagus, length	—	0.91 × 0.098	0.67	0.37–0.38 (0.375)^*∗∗*^
Glandular oesophagus, length	—	2.55 × 0.154	2.21	1.6–2.4 (2)^*∗∗*^
Tail, length	0.416–0.488	0.56	—	0.43^*∗*^
Long spicule (lsp)	1.6–1.8	2.4	1.48–1.74 (1.60)	1.4–1.6 (1.44)
The ratio between lsp length and body length	—	0.17	0.16	0.11
short spicule (ssp)	0.2–0.22	0.18	0.22–0.26 (0.22)	0.5–0.8 (0.62)
The ratio between ssp length and body length	—	0.01	0.02	0.04
Pairs of postcloacal Papillae	6	3	5	5
*Female*	—	*n* = 2	*n* = 5	*n* = 5
Body, length (mm)	15–22	24-25	9.7–23.09 (15.36)	16–20 (17.5)
Body, width (mm)	0.4–0.65	0.46–0.5	0.40	0.35–0.45 (0.39)
Cordons, length	10–15	—	—	12.5–13.8 (12.96)
Buccal cavity, length	—	0.33 × 0.047–0.057	—	0.23 × 0.028^*∗*^
Muscular oesophagus, length	—	1.26–1.3	0.82	0.29–0.75 (0.45)^*∗∗∗*^
Glandular oesophagus, length	—	4.2–4.5	2.83	2.1–2.7 (2.3)^*∗∗∗*^
Tail, length	0.42–0.59	0.28	0.33	0.44^*∗*^
Egg (*μ*m)	40 × 27	39–45 × 24–26	36 × 22	51.25 × 29.5

^*∗*^Measurements from one male and female only.

^*∗∗*^Measurements from two males only.

^*∗∗∗*^Measurements from three females only.
